# Association of Pyoderma Gangrenosum, Acne, and Suppurative Hidradenitis (PASH) Shares Genetic and Cytokine Profiles With Other Autoinflammatory Diseases: Erratum

**DOI:** 10.1097/01.md.0000464213.86167.ec

**Published:** 2015-02-27

In the article “Association of Pyoderma Gangrenosum, Acne, and Suppurative Hidradenitis (PASH) Shares Genetic and Cytokine Profiles With Other Autoinflammatory Diseases”,^1^ which appeared in Volume 93, Issue 27 of *Medicine*, one of Orietta M. Borghi's affiliations was omitted. The article should have stated that Orietta M. Borghi is associated with the IRCCS Istituto Auxologico Italiano, Milano, Italy as well as the Dipartimento di Scienze Cliniche e di Comunità, Università di Milano. The article has since been corrected online.
